# Patent foramen ovale leads to transient loss of consciousness: case reports

**DOI:** 10.3389/fnins.2026.1747877

**Published:** 2026-03-18

**Authors:** Yue Liu, Cui Gao, Weili Jing, Xintao Tian, Cuiping Li, Xinrui Cheng, Xi Rong, Jinghui Song, Shaonan Yang

**Affiliations:** 1 Department of Neurology, The Affiliated Hospital of Qingdao University, Qingdao, Shandong, China; 2 Department of Critical Care Medicine, The Affiliated Hospital of Qingdao University, Qingdao, Shandong, China; 3 Department of Urology, The Affiliated Hospital of Qingdao University, Qingdao, Shandong, China

**Keywords:** paradoxical embolism, PFO, syncope, TLOC, transcranial Doppler ultrasound bubble test

## Abstract

Patent foramen ovale (PFO) is a congenital heart condition with a prevalence of 20%–35% in the general population. It is associated with various paroxysmal neurological seizures. We report four patients who presented to our hospital with transient loss of consciousness (TLOC). They were diagnosed with PFO after a comprehensive examination. In this article, we discuss the mechanism related to PFO and TLOC, which provides a new clinical perspective for the diagnosis of TLOC.

## Introduction

Transient loss of consciousness (TLOC) can be categorized into two types: syncope and non-syncope (Puppala et al., 2014). Syncope is defined as TLOC resulting from transient global cerebral hypoperfusion, which is characterized by a sudden onset, short duration, and spontaneous complete recovery (Costantino et al., 2016). Syncope can be caused by many conditions including cardiogenic syncope, orthostatic hypotension, neurally mediated syncope, and cerebral syncope (Bassetti, 2014). Cardiogenic syncope is typically caused by arrhythmia or organic cardiovascular diseases (Chen-Scarabelli and Scarabelli, 2004). Neurally mediated syncope includes carotid sinus hypersensitivity and vasovagal syncope. This syncope is often triggered by predisposing factors such as coughing, urination, and defecation (Gauer, 2011). Cerebral syncope can be caused by transient ischemic attack or subclavian steal syndrome. In addition, TLOC caused by epileptic seizure, concussions, hypoglycemia, and other causes is classified as non-syncope (Koene et al., 2017). What other causes should we consider when the medical history and clinical examination of the above causes of TLOC do not find obvious abnormalities? Here we provide a new clinical approach.

Patent foramen ovale (PFO) is a common anatomical variation, accounting for about 20%–35% of the population (Calvert et al., 2011). PFO plays an important role in the occurrence and development of various diseases, such as cryptogenic stroke (Lee et al., 2018), migraine (Trabattoni et al., 2022), decompression sickness (Bove, 1998), platypnea–orthodeoxia syndrome (Blanche et al., 2013), obstructive sleep apnea syndrome and so on (Hoole et al., 2017). In recent years, TLOC caused by PFO has gradually received extensive attention from clinicians (Wêglarz et al., 2024). Although TLOC is benign in most patients,

recurrent episodes of TLOC can affect quality of life and lead to associated injuries. It is important to identify the risk factors for TLOC and take effective preventive measures to reduce its episodes (Zou et al., 2024). Here, we introduce four cases of TLOC caused by PFO.

## Case reports

### Case 1

**FIGURE 1:**
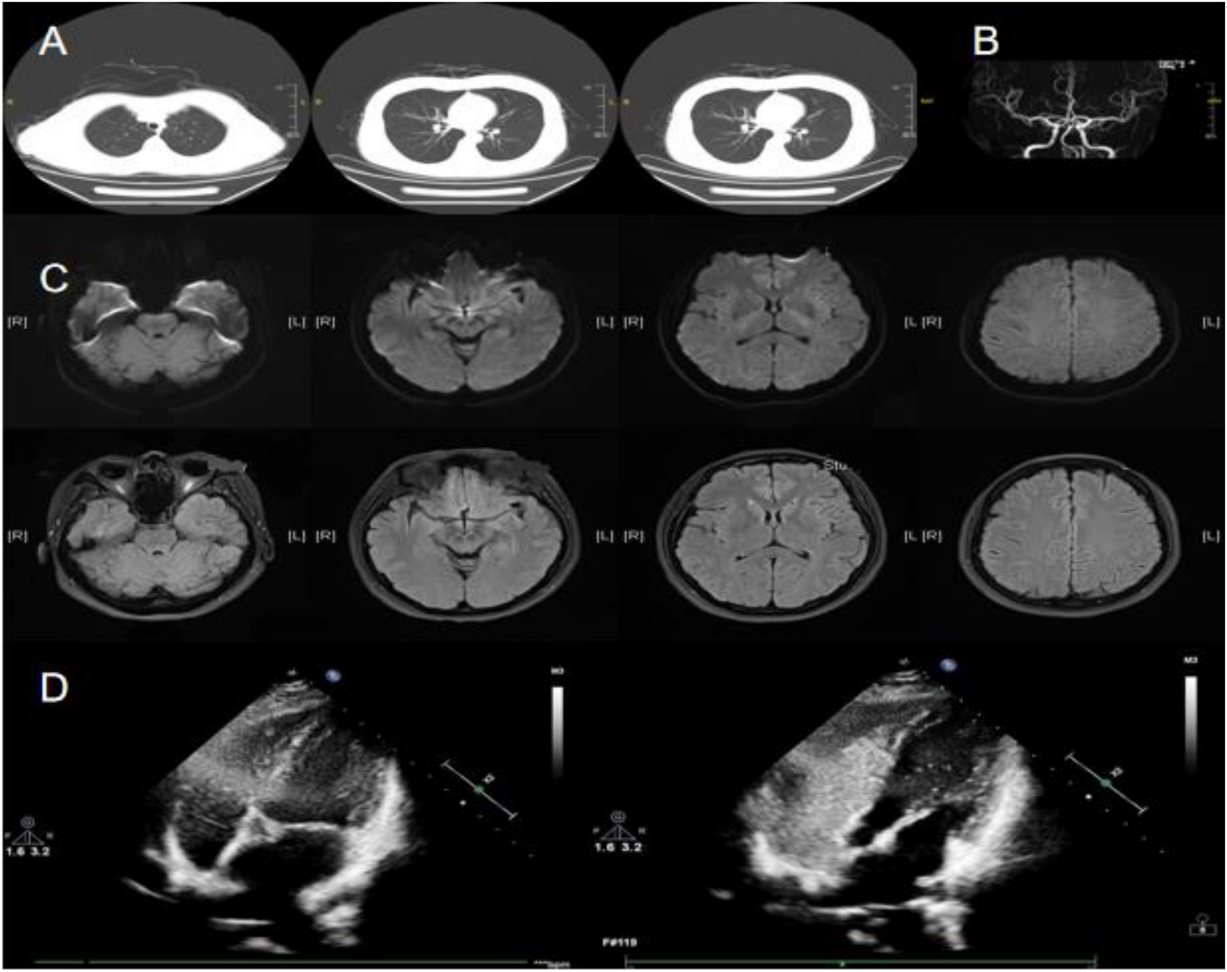
Main examination findings for case 1: **(A)** chest CT; **(B)** cranial MRA; **(C)** cranial MRI; **(D)** right heart catheterization imaging.

A 38-years-old woman, previously healthy, was admitted due to headache and TLOC for more than 10 years. Physical examination was unremarkable. Hematological examination, including complete blood count (CBC), liver and kidney function tests, electrolytes, blood profile, coagulation function test, homocysteine, troponin, and B-type natriuretic peptide (BNP) were all in the normal range. Chest CT showed a small nodule in the lower lobe of the right lung (Figure 1A). Brain MRI and MRA showed no significant abnormalities (Figures 1B, C). Cervical vascular ultrasound, 24-h video EEG, and echocardiography also showed no abnormalities. A 24-h holter electrocardiogram showed sinus rhythm. Right heart catheterization confirmed a PFO with significant right-to-left shunting (grade 3, characterized by many microbubbles) (Figure 1D). The patient decided to undergo PFO closure due to long-term headaches, and the headache did not recur after surgery. After long-term follow-up, we discovered that the patient did not experience recurrence of TLOC. This suggests that PFO may be a contributing factor in TLOC.

### Case 2

**FIGURE 2:**
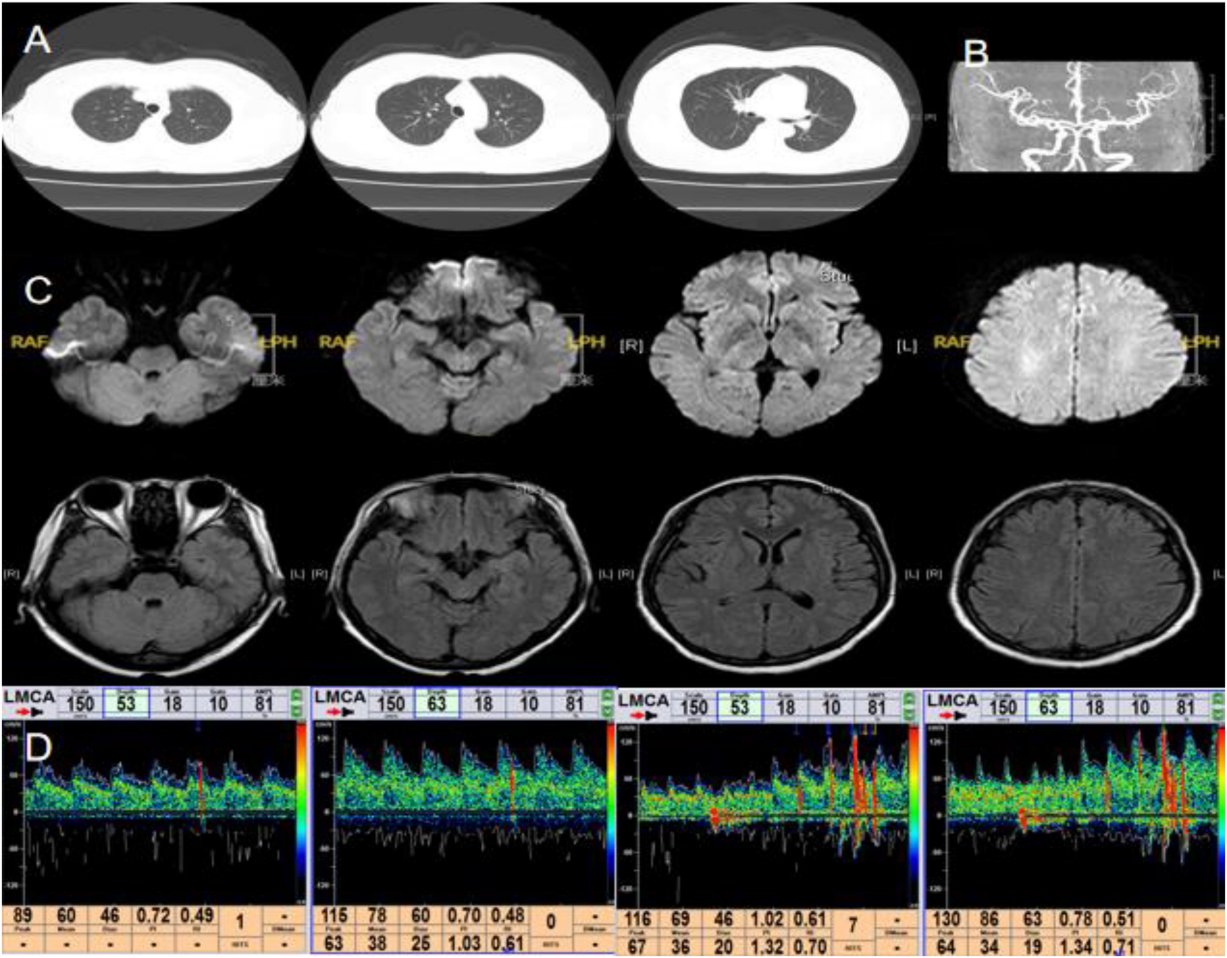
Main examination findings for case 2: **(A)** chest CT; **(B)** cranial MRA; **(C)** cranial MRI; **(D)** transcranial Doppler ultrasound bubble test.

A 44-years-old female underwent PFO closure 10 years ago due to a heart murmur. Recently, she presented to our hospital with sudden TLOC. Physical examination revealed no positive findings. Hematological examination showed hyperlipidemia, with total cholesterol of 5.96 mmol/L and LDL of 4.4 mmol/L. Routine hematological parameters, including CBC, liver and kidney function tests, electrolytes, coagulation function test, homocysteine, troponin and BNP were in the normal range. Chest CT showed postoperative changes in the atrial septum (Figure 2A). Brain MRI and MRA revealed no significant abnormalities (Figures 2B, C). Cervical vascular ultrasound and 24-h video EEG also showed no abnormalities. Cardiac ultrasound showed postoperative changes in the atrial septum. A 24-h holter electrocardiogram showed sinus rhythm. No obvious abnormalities were found in the above results. Considering the relevant clinical manifestations of the case 1, we wondered whether the patient still had right-to-left shunting. We decided to conduct the transcranial Doppler ultrasound bubble test again. The result revealed 2 microemboli signals at rest and 10 microemboli signals after Valsalva maneuver (Figure 2D), indicating recurrent right-to-left shunting following PFO closure. Considering the patient’s history of PFO closure, we initiated antiplatelet therapy with aspirin. After 1 year of follow-up, the patient has not experienced TLOC.

Based on the clinical symptoms of the two patients, we hypothesized: Could PFO contribute to the occurrence of TLOC? Subsequently, we collected another two cases of PFO complicated by TLOC (Table 1).

**TABLE 1:** Clinicalcharacteristics and test results of patients Case 3 and Case 4.

	Case 3	Case 4
Age	66	50
Gender	Male	Female
Past medical history	Hypertension and sudden deafness in his right ear.	Two similar episodes of syncope.
Triggering factors	Lifting heavy objects	Exercise
Physical examination	No positive physical signs	No positive physical signs
Blood test	D-dimer: 760 ng/ml, CBC, blood glucose, electrolytes, liver and kidney function tests, lipid profile, troponin, BNP, and homocysteine were all in the normal range.	CBC, blood glucose, electrolytes, liver and kidney function tests, lipid profile, troponin, BNP, and homocysteine were all in the normal range.
Chest CT	Normal	Normal
Cranial MR	Multiple cerebral softening lesions in the pons, right basal ganglia, and left corona radiata region	Normal
Cranial MRA	Cerebral atherosclerosis (AS)	Cerebral AS
Carotid CTA	AS and mild stenosis of part of blood vessels	–
Cervical vascular ultrasound	–	Localized thickening of the intima-media in both common carotid arteries.
24-h video EEG	Normal	Normal
Cardiac ultrasound	Left ventricular septal hypertrophy and degenerative changes of the aortic valve	Normal
Coronary CTA	Mild atherosclerotic changes in the coronary arteries	–
24-h holter electrocardiogram	Sinus rhythm	Sinus rhythm
Transcranial Doppler ultrasound bubble test	Microemboli passed both at rest and during Valsalva maneuver, with a total count greater than 25, and more during the Valsalva maneuver.	No microemboli detected at rest, but 23 microembolic signals were observed during the Valsalva maneuver.
Transesophageal echocardiography/right cardiac catheterization	Transesophageal echocardiography: PFO	Right cardiac catheterization: PFO, with a moderate amount of contrast microbubbles visible in the left atrium after provocative maneuver.
Treatment	Aspirin	Aspirin
Table	Figure 3	Figure 4

“–” indicates that the relevant examination was not performed.

**FIGURE 3:**
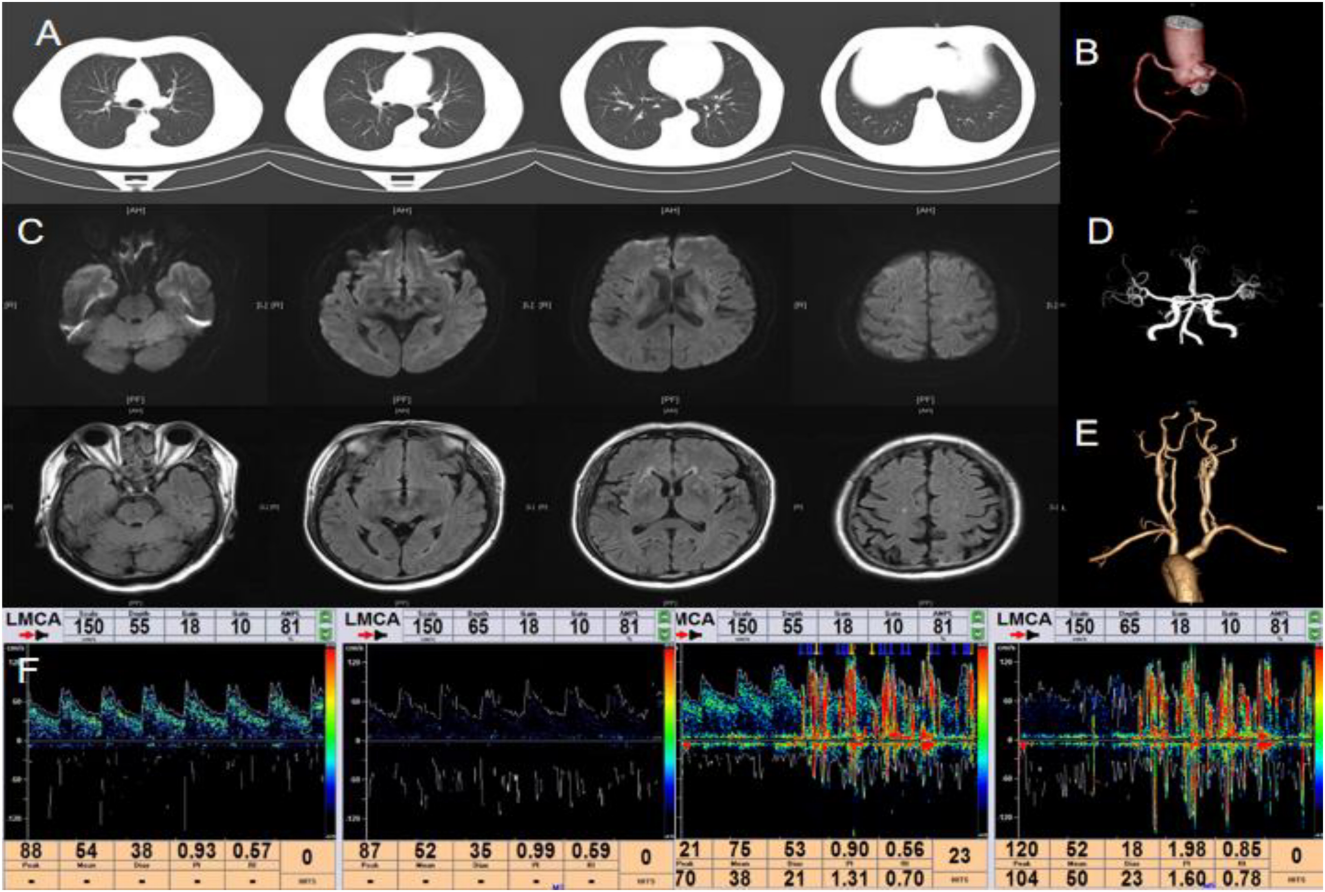
Main examination findings for case 3: **(A)** chest CT; **(B)** coronary CTA; **(C)** cranial MRI; **(D)** cranial MRA; **(E)** carotid CTA; **(F)** transcranial Doppler ultrasound bubble test.

**FIGURE 4:**
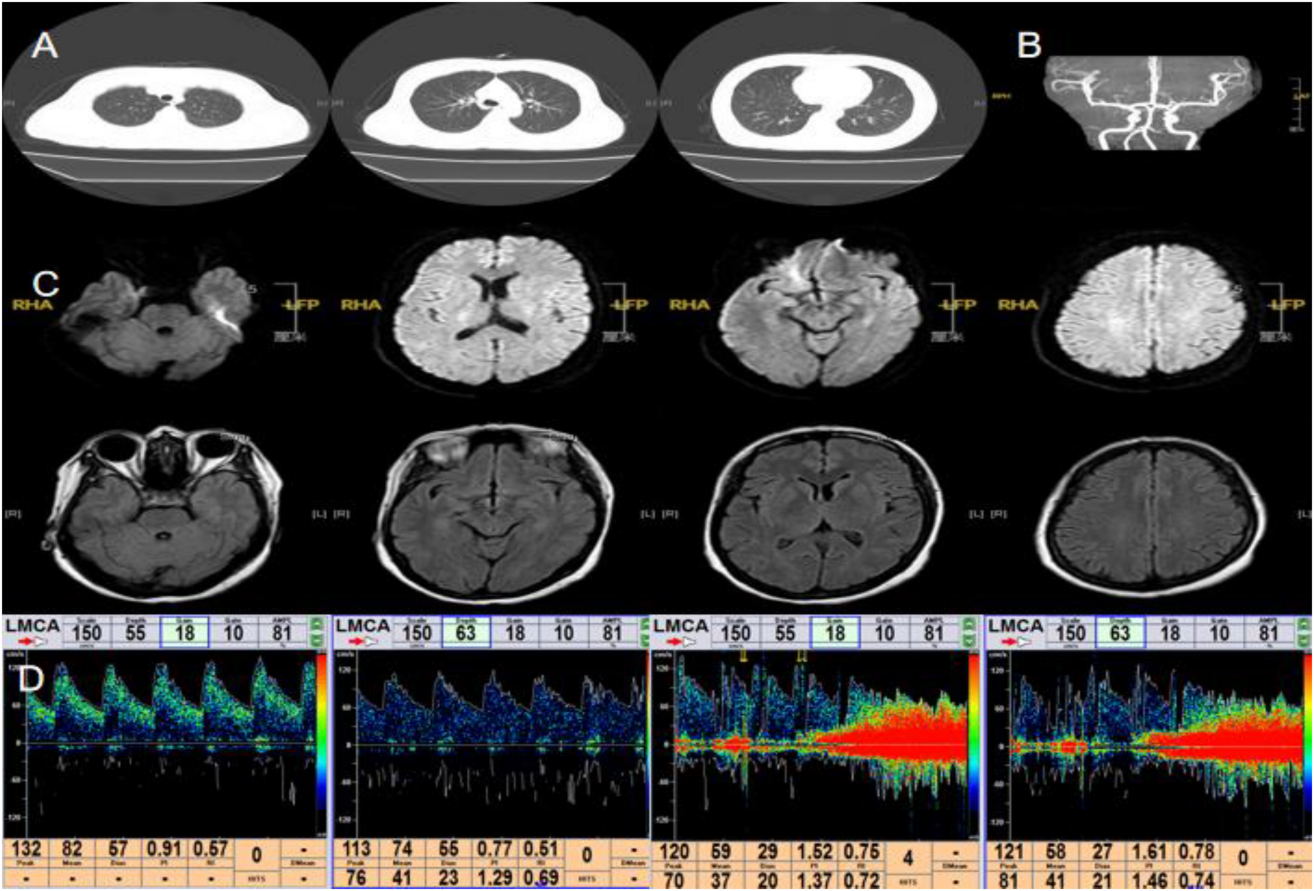
Main examination findings for case 4: **(A)** chest CT; **(B)** cranial MRA; **(C)** cranial MRI; **(D)** transcranial Doppler ultrasound bubble test.

## Discussion

The foramen ovale is a normal fetal structure that allows oxygenated blood returning to the right atrium to reach the brain and vital organs directly (Abdelghani et al., 2019; Mojadidi et al., 2015). It is formed by the incomplete fusion of the primary and secondary septa (Abdelghani et al., 2019). After birth, the drop in pulmonary pressure causes left atrial pressure to exceed right atrial pressure, pushing the septum primum against the septum secundum and leading to functional closure of the foramen ovale (Kjeld et al., 2018; Wessler and Kent, 2015). This closure is usually completed before the age of two (Barrios et al., 2017). However, PFO still occurs in 20%–35% of the population (Beelke et al., 2003). PFO is associated with the development of various diseases. In this article, we mainly discuss TLOC caused by PFO.

There are two mechanisms of TLOC caused by PFO: first, abnormal embolism mechanism. Paradoxical embolism refers to the process where emboli from the venous system enter the arterial system through an abnormal cardiac pathway (Kottoor and Arora, 2018). Under normal physiological conditions, left atrial pressure exceeds the right, resulting in a pressure gradient that prevents or minimizes shunting through a PFO (Guo et al., 2007).

During some Valsalva maneuvers, such as coughing, sneezing, squatting, or weightlifting, the pressure of right atrial will increase, resulting in right-to-left shunting (Beelke et al., 2003). At the same time, the region of foramen ovale is prone to persistent impact from the inferior vena cava, and long-term blood flow impingement may lead to local tissue microulcers (Zhu et al., 2024). In addition, the hemodynamic changes caused by PFO may promote activation of coagulation factors and adhesion of blood cells (Elgendy et al., 2020). Under conditions that satisfy Virchow’s triad, the formation of *in situ* microthrombus is likely to occur. Thrombi or vasoactive substances from the venous system can enter the intracranial arterial system through right-to-left shunting, causing arterial spasms and transient cerebral ischemia, further inducing syncope (Kizer and Devereux, 2005; Ravi et al., 2024). When the microemboli enter the cerebral cortex via the bloodstream, the cortex is suppressed, and the patient will also experience syncope (Kelley and Kelley, 2021).

The second mechanism is hypoxia. Deoxygenated venous blood from the right atrium directly enters the left atrium through a PFO and mixes with oxygenated arterial blood to cause hypoxia in the body (Dehnert et al., 2007). When patients are in a hypoxic environment for a long time, pulmonary blood vessel constriction leads to elevated pulmonary blood vessel pressure. The elevated right atrial pressure can lead to the opening of the foramen ovale (Moses et al., 2015). There is a vicious cycle between hypoxia and PFO.

Case 1 had no further TLOC episodes during the year following PFO closure. However, case 2 presented with TLOC after recanalization of the previously closed foramen ovale. These two cases illustrate the potential causal relationship between PFO and TLOC from complementary perspectives. This also prompted our subsequent attention to the relationship between PFO and TLOC. In case 3 and 4, the patients experienced TLOC during physical exertion (e.g., heavy lifting or exercise). The increased right atrial pressure from these activities likely caused transient right-to-left shunting through a PFO, enabling microemboli to enter the arterial system and reach the cerebral vasculature. Additionally, case 4 experienced TLOC during exercise. In addition to the above mentioned possible induction of Valsava maneuver, there may also be a possible mechanism related to hypoxia. In case 4, TLOC occurred during physical exertion. This may involve not only the Valsalva-related mechanism described above but also a hypoxic component. Hayashida et al. (2001) found that embolism caused by PFO mainly occurred in cerebral cortex and vertebrobasilar artery regions through radionuclide venography. Previous deafness in case 3 could also be attributed to embolism in the vertebrobasilar artery. This is mainly because the anterior inferior cerebellar artery, which originates from the vertebrobasilar artery, is the main blood vessel supplying the inner ear (Baldi et al., 2012). The diameter of the PFO is an independent risk factor for ischemic stroke (Beelke et al., 2003). PFO diameters ranging from 1 to 19 mm (mean 4.9 mm) allow emboli to pass through the venous system. These emboli are often large enough to occlude the middle cerebral artery or its cortical branches upon reaching the cerebral circulation (Abdelghani et al., 2019; Yan et al., 2023). The multiple cerebral softening lesions observed in Case 3 are also associated with these microemboli.

Currently, international guidelines suggest that closure of PFO is superior to medical therapy alone for PFO-related stroke (Lee et al., 2018; Mas et al., 2017; Søndergaard et al., 2017; Saver et al., 2017). For patients with TLOC and PFO, both PFO closure and medical therapy are effective, but there is no relevant expert consensus. Further research is needed to determine whether PFO closure is superior to medical therapy in these patients.

This case report aims to provide a new diagnostic approach for TLOC in clinical practice. However, due to the small sample size of this study, its findings have certain limitations in terms of generalizability. Furthermore, due to the lack of direct monitoring data during the episodes and advanced imaging evidence, the exact pathophysiological mechanism of this condition requires further investigation and confirmation.

## Conclusion

At present, TLOC caused by PFO has not been concerned by the majority of neurologists. Here we provide a new clinical idea for clinicians. Meanwhile, large sample clinical studies are still needed to determine whether PFO closure is superior to medical therapy in patients with unexplained TLOC and PFO.

## Data Availability

The original contributions presented in this study are included in this article/supplementary material, further inquiries can be directed to the corresponding authors.
